# Psychology as a Natural Science Applied to the Solution of Occult Psychic Phenomena

**Published:** 1889-07

**Authors:** 


**Psychology as a Natural Science Applied to the Solution of Occult Psychic Phenomena.** By C. G. Raue, M. D. Philadelphia: Porter & Coates. 1889. Octavo; cloth; pp. 541.

This book, as its title indicates, deals with the intangible and abstruse subjects which defy proof.

The author writes from a standpoint opposed to the physical school. According to his view, the processes of the mind should not be studied by physical laws, as they are not controlled by them. A firm believer in the mystical, the author has occasion to show why houses may be haunted by the spirits of the dead, and why the mind can act on persons removed from them by terrestrial distances, because the soul does not require space.

His description of the soul is interesting: "The soul consists of that organized system of immaterial forces by which it projects itself, on the one hand, into the material world. The soul consists, therefore, of an immaterial, nervous, respiratory, osculatory, generative, muscular, bony, cutaneous system; has eyes, ears, nose, mouth, and all the organs, in every particular, as expressed materially in the human body. On the other hand, by its higher immaterial forces, the higher senses, it develops into all those conscious modifications of which we have been treating in this work as cognitions, conations, and feelings, and all their wonderful combinations."

He deals with the soul as if he were perfectly familiar with it,—its habits, and its future existence. On page 376 he says "that the soul constantly increases in internal strength from birth to death."

He proves, satisfactorily to himself, that the soul continues to exist after death. The constant tendency of the author is to explain and argue from a spiritualist standpoint.

In his treatment of the subject of hypnotism, he ignores the work of Bernheim.

The book is interesting, as it shows us the extreme psychological method adopted by a school of thinkers at present in the minority. We ourselves prefer to explain such phenomena as are explainable by physical laws. Instead of accepting, as he does, the stories of second sight, we are inclined to doubt the record. We must acknowledge that we belong to the class to whom he refers as wishing to see ghosts before we believe in them.

It is a satisfaction to know that some one is positive as to what follows after death. On page 539 we read: "What follows after the separation of soul and body? Answer, Continued evolution." He says further: "We shall have to submit to these facts, and, consequently, the possibility of an intercourse between departed spirits and this corporeal world is likewise established."

The book is evidently the work of a student, and is carefully written. Cases of rare interest are cited. The typographical beauty of the book adds pleasure to its perusal.

